# A MEMS Micro-g Capacitive Accelerometer Based on Through-Silicon-Wafer-Etching Process

**DOI:** 10.3390/mi10060380

**Published:** 2019-06-07

**Authors:** Kang Rao, Xiaoli Wei, Shaolin Zhang, Mengqi Zhang, Chenyuan Hu, Huafeng Liu, Liang-Cheng Tu

**Affiliations:** 1 MOE Key Laboratory of Fundamental Physical Quantities Measurement, School of Physic, Huazhong University of Science and Technology, Wuhan 430074, China; raokang@hust.edu.cn (K.R.); weixiaoli@hust.edu.cn (X.W.); zhangshaolin@hust.edu.cn (S.Z.); zhangmengqi@hust.edu.cn (M.Z.); chenyuanhu@hust.edu.cn (C.H.); 2 Institute of Geophysics and PGMF, Huazhong University of Science and Technology, Wuhan 430074, China

**Keywords:** MEMS, microfabrication, accelerometer, micro-g, capacitance displacement transducer, through-silicon-wafer-etching

## Abstract

This paper presents a micromachined micro-g capacitive accelerometer with a silicon-based spring-mass sensing element. The displacement changes of the proof mass are sensed by an area-variation-based capacitive displacement transducer that is formed by the matching electrodes on both the movable proof mass die and the glass cover plate through the flip-chip packaging. In order to implement a high-performance accelerometer, several technologies are applied: the through-silicon-wafer-etching process is used to increase the weight of proof mass for lower thermal noise, connection beams are used to reduce the cross-sensitivity, and the periodic array area-variation capacitive displacement transducer is applied to increase the displacement-to-capacitance gain. The accelerometer prototype is fabricated and characterized, demonstrating a scale factor of 510 mV/g, a noise floor of 2 µg/Hz^1/2^ at 100 Hz, and a bias instability of 4 µg at an averaging time of 1 s. Experimental results suggest that the proposed MEMS capacitive accelerometer is promising to be used for inertial navigation, structural health monitoring, and tilt measurement applications.

## 1. Introduction

Microelectromechanical systems (MEMS) accelerometers have been widely used in various application fields, such as consumer electronics, automobiles, medical, structural health monitoring, and inertial navigation [1,2,3,4]. According to the displacement or force transduction mechanisms, MEMS accelerometers can be categorized as different types, including piezoelectric [5,6], capacitive [7,8], piezoresistive [9,10], tunneling [11,12], resonant [13,14,15,16], optical [17,18], thermal [19,20], and electromagnetic [21,22] accelerometers. Capacitive transduction is one of the most commonly used technologies for high-performance MEMS accelerometers since it takes advantage of simple structure, low noise, low power consumption, cost-effectiveness, and reliability [4]. Most of the state-of-the-art capacitive MEMS accelerometers use sandwich [23] or comb-finger [24] gap-variation based capacitive displacement transducers. However, due to the nonlinear capacitance-to-displacement relationship [25] and pull-in effect [26], gap-variation based capacitive accelerometers generally require closed-loop control, which increases circuit complexity and power consumption. Even though area-variation based capacitive displacement transducers have intrinsically good capacitance-to-displacement linearity and large travel range for open-loop operation, the MEMS inertial sensors based on area-variation technology [27,28] are less commonly used than the gap-variation based ones. One of the main reasons is that area-variation capacitive accelerometers are difficult to be fabricated by either bulk silicon wet etching or silicon-on-insulator-based processes that are widely used for fabricating high-performance gap-variation based MEMS accelerometers. In this paper, we introduce a through-silicon-wafer-etching process that offers a solution of fabricating high-performance accelerometers by keeping both wafer-thick bulk proof mass and the area-variation capacitive displacement transducer. Based on this fabrication technology, a high-performance open-loop MEMS capacitive accelerometer has been developed and characterized.

## 2. Materials and Methods 

**Figure 1:**
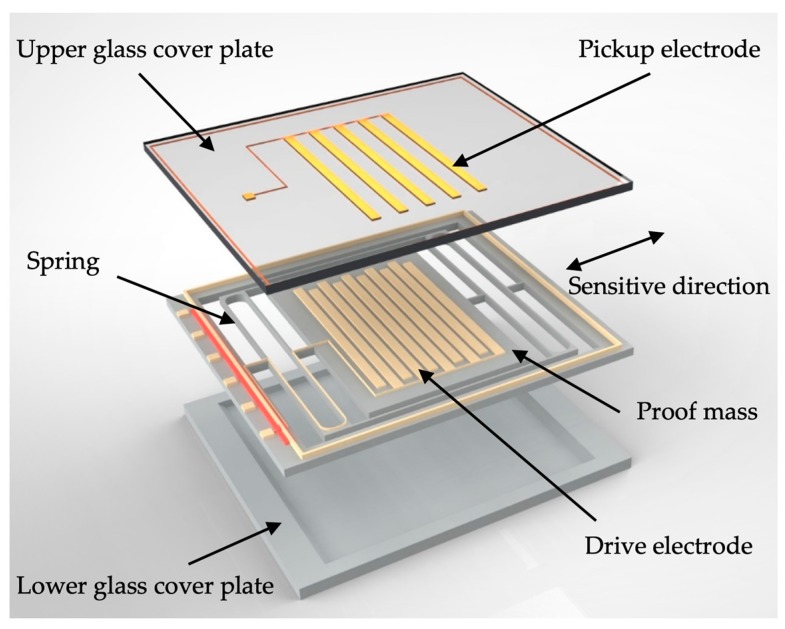
Schematic of the proposed MEMS accelerometer.

The proposed MEMS accelerometer consists of a silicon-based acceleration-sensitive spring-mass structure which is sandwiched by the upper and lower glass cover plates, as illustrated in Figure 1. The in-plane motion of the proof mass is sensed capacitively between the array of parallel-plate electrodes on the proof mass and the matching array of electrodes on the upper glass plate which is separated by a fixed gap above the proof mass.

### 2.1. Spring-Mass Structure Design

**Figure 2:**
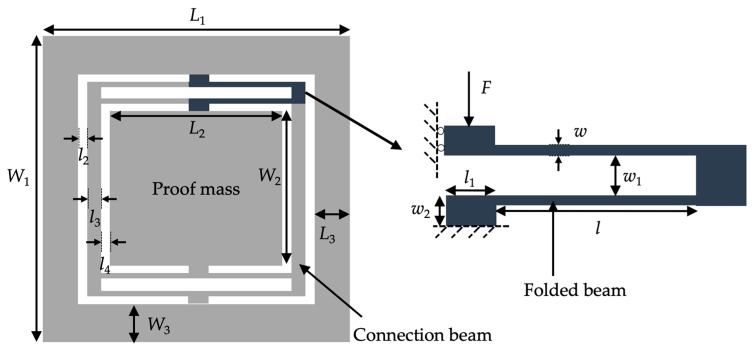
Structure of the spring-mass system.

The spring-mass system of the proposed MEMS accelerometer is mainly composed of the suspension, the proof mass, and the sensor frame, as shown in Figure 2. The suspension has symmetrical configurations that each compliant component is a folded spring beam and four identical compliant components are connected in parallel to suspend the central proof mass on the sensor frame. For a single cantilever beam with one end fixed and the other end guided, if the force *F* is applied on the guided end along the sensitive X-axis, the displacement along *X*-axis is:

(1) $$\Delta x=\frac{{Fl}^{3}}{12{EI}_{x}},$$

where *l* is the length of the cantilever beam, *E* is Young’s modulus, *I_x_* is the area moment of inertia about *X*-axis and it is equal to 1/12*w^3^t*, *w* is the spring width and *t* is the wafer thickness. Thus, the stiffness of the guided cantilever beam along *X*-axis is:

(2) $$k_{1}=\frac{F}{\Delta x}=\frac{12EI_{x}}{l^{3}}.$$

The folded spring beam is equivalent to two cantilevers connected in series, doubling the compliance; therefore, the stiffness of the folded beam *k*_2_ is a half of a cantilever *k*_1_, having

(3) $$k_{2}=\frac{k_{1}}{2}\frac{6EI_{x}}{l^{3}}.$$

Since four identical folded beams work together in parallel, the overall suspension stiffness of the accelerometer along the sensitive axis can be presented as:

(4) $$k_{x}=4k_{2}=\frac{24EI_{x}}{l^{3}}.$$

The resonant frequency of the spring-mass system is:

(5) $$\omega_{0}=\sqrt{\frac{k_{x}}{m}}.$$

The thermal noise equivalent acceleration (TNEA) of the spring-mass accelerometer is:

(6) $$\mathrm{TNEA}=\sqrt{\frac{4k_{B}T\omega_{0}}{mQ}}.$$

According to Equations (5) and (6), in order to achieve a lower noise floor under a certain temperature and pressure, the proof mass should be increased and the suspension stiffness should be decreased. However, increasing the proof mass is not cost-efficient, while decreasing the stiffness *k_x_* is a better approach. According to Equation (4), the stiffness can be lowered down by increasing the beam length or minimizing the area moment of inertia. However, when designing the mechanical structure of the accelerometer, apart from the noise floor, the work range and bandwidth should also be considered. There are trade-offs that the greater the stiffness, the greater the range and bandwidth, but the larger the noise floor. In addition, since the proposed single-axis accelerometer utilizes the area-variation based capacitive sensing technology, the gap between the matching electrodes should ideally be constant. However, in practical, accelerations along arbitrary direction exerted on the accelerometer can result in gap changes thus introduce cross-sensitivities. Therefore, the design rationale is to obtain a smaller stiffness along *X*-axis and larger stiffnesses for other axes, having the rejection ratios of cross-axis resonant frequencies to the fundamental frequency to be as large as possible. In order to increase the rejection ratios, two connection beams are introduced into the suspension system to bridge the folded beams on both sides of the proof mass together.

The design target of the proposed accelerometer is to have a work range of at least 20 g, a −3 dB bandwidth of at least 200 Hz, and a noise floor of lower than 10 μg/Hz^1/2^ at 100 Hz. According to the target specifications, the design parameters of the spring-mass structure are selected and specified in Table 1.

**Figure 3:**
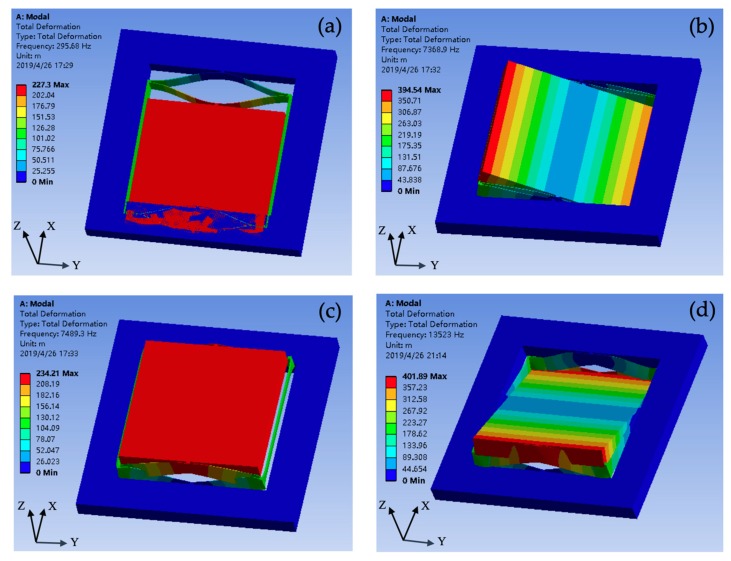
Finite element analysis (FEA) simulation results of the eigenmode: (**a**) fundamental translational mode X; (**b**) rotational mode α about *X*-axis; (**c**) translational mode Z; (**d**) rotational mode β about *Y*-axis.

A finite element analysis (FEA) software ANSYS was used to simulate the eigenmode of the spring-mass structure of the proposed accelerometer. The simulation results are shown in Figure 3. Another simulation about the spring-mass structure without connecting beams was also conducted. The simulation results are listed in Table 2. It can be seen that the rejection ratios of the structure with connection beams are 30%–77% larger than those without connecting beams, indicating that the optimization design reduces the cross-sensitivity from 0.48% to 0.16%.

### 2.2. Area-Variation Capacitive Displacement Transducer Design

**Figure 4:**
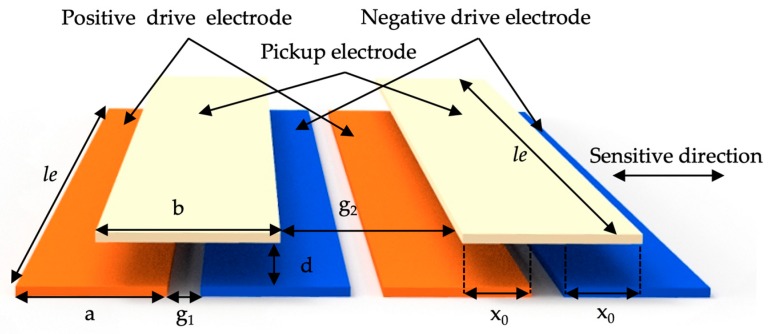
Schematic of the area-variation capacitive displacement transducer configuration.

The spring-mass system of the accelerometer transforms the external accelerations to displacements of the proof mass. In order to measure accelerations, it is necessary to convert the displacement into other measurable quantities. By applying capacitive displacement sensing technology, the displacement can be transduced into capacitance changes which can then be converted to voltage or current through the signal conditioning circuit. In this case, the capacitive accelerometer performs as an acceleration ratio-meter. An area-variation periodic array capacitive displacement transducer is introduced with the schematic illustrated in Figure 4. Each set of the periodic array capacitive transducer consists of two drive electrodes plated on the movable proof mass and one pickup electrode plated on the upper glass cover plate with a gap of *d*. The overlapping length of the drive and pickup electrodes is *l_e_*. The width of both drive electrodes is defined as *a* and the separation between each drive electrode is *g*_1_. While the width of the pickup electrode is *b* and the separation between each other is *g*_2_. When the electrodes pairs are located in the null position where the pickup electrode is right in the middle of two drive electrodes, the overlapping width of the pickup electrode to each drive electrode is *x_0_*. In this case, the sensing capacitors *C*_1_, constructed by the positive drive electrode and the pickup electrode, and *C*_2_, constructed by the negative drive electrode and the pickup electrode, have the same capacitance:

(7) $$C_{1}\;=\;C_{2}\;=\;C_{0}\;=\;\frac{\varepsilon l_{e}x_{0}}{d},$$

where *C*_0_ is the nominal capacitance of one period and *ε* is the permittivity. When there is a relative displacement Δ*x* between the proof mass and the upper cover plate along the sensitive direction, the overlapping areas of *C*_1_ and *C*_2_ change in opposite, having

(8) $$C_{1}\;=\;\frac{\varepsilon l_{e}(x_{0}+\Delta x)}{d},\;C_{2}\;=\;\frac{\varepsilon l_{e}(x_{0}-\Delta x)}{d}.$$

Thus, the capacitance difference of these two capacitors can be obtained,

(9) $$\Delta C\;=\;C_{1}\;-\;C_{2}\;=\;\frac{2\varepsilon l_{e}\Delta x}{d}.$$

It can be seen from Equation (9) that the displacement variation caused by accelerations can be converted into capacitance change by the area-variation capacitive displacement transducer with differential configuration, thus realizing capacitive displacement sensing.

In the actual device design, in order to increase the gain of displacement-to-capacitance, arrays with N periods of the three-electrode differential configuration unit are applied. The capacitance change of periodic transducer in terms of the relative displacement is N times of the single three-electrode unit. Therefore, the displacement-to-capacitance gain is significantly increased. In order to make the displacement-to-capacitance gain as large as possible, the electrodes take the entire surface area of the proof mass to maximize the periods. In addition, the periodic width should match the travel range of the proof mass to ensure the movement within a single period. Considering the spring-mass structure and the accelerometer performance requirements, the design parameters of the periodic array area-variation capacitive displacement transducer are selected and listed in Table 3. 

**Figure 5:**
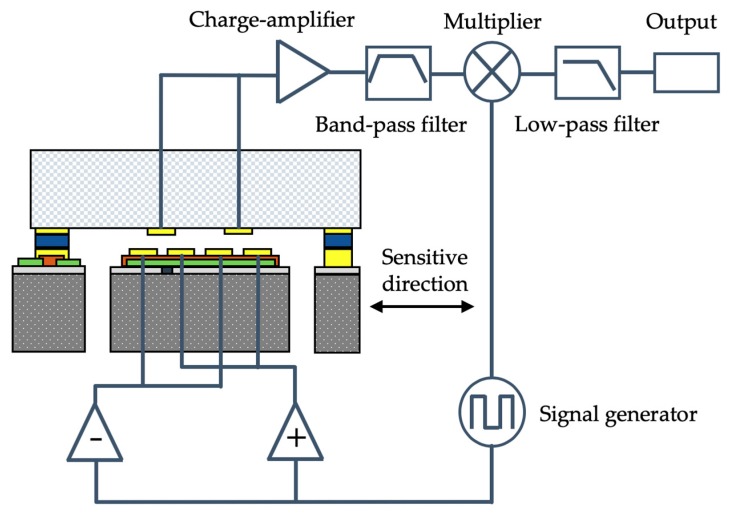
Schematic of the capacitive displacement transducer and the conditioning circuit.

The schematic of the conditioning circuit for the periodic array area-variation capacitive displacement transducer is illustrated in Figure 5. The carrier signal with a frequency of 100 kHz is applied both on the positive drive electrode and the negative drive electrode through a buffer and an inverter, respectively. Therefore, the positive and negative electrodes have a constant phase difference of 180° but the same amplitude. When the proof mass moves along the sensitive direction, the capacitance of periodic array area-variation capacitive displacement transducer changes; therefore, the charges flow in/out through a charge amplifier with the output in voltage, realizing the C-V conversion. Then, the voltage signal passes through a band-pass filter to have a narrow-band signal for lowering down interferences. The synchronous demodulation technology is applied to obtain the weak and low-frequency capacitance variation signal by a multiplier and the following low-pass filter. Since the power supply of amplifiers is ±12 V, the analog output voltage range is at least ±10 V.

## 3. Fabrication Process

**Figure 6:**
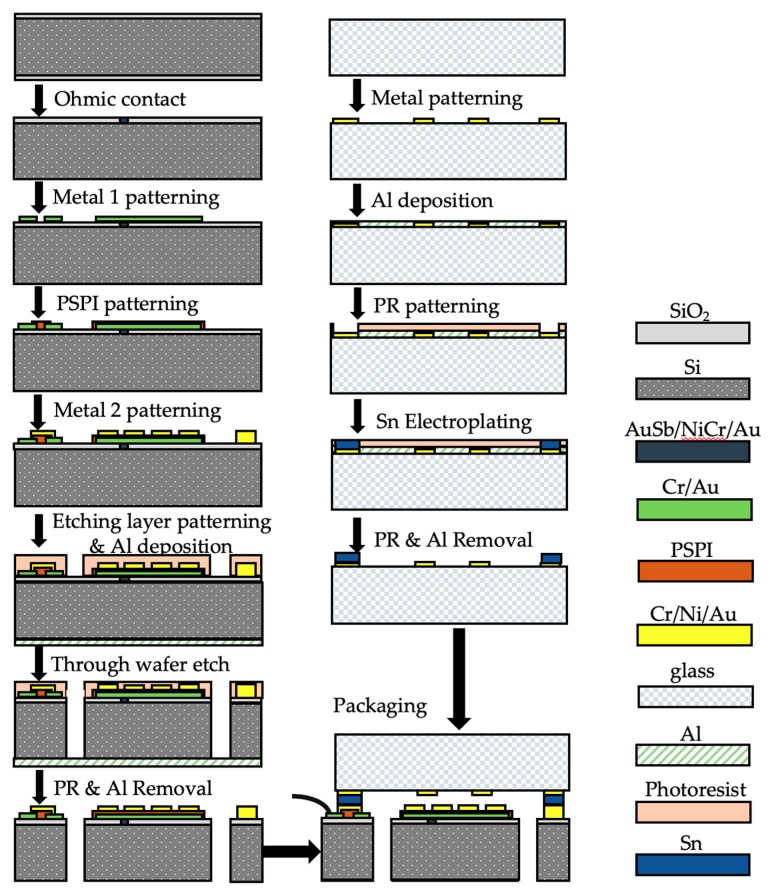
The microfabrication process flow of the proposed MEMS accelerometer.

The functional features of the proposed accelerometer are based on single-crystal silicon substrate and Borofloat 33 glass substrate which require to be fabricated separately. The silicon substrate includes the spring-mass movable structure, the drive electrodes of the capacitive transducer, and the packaging and wire-bonding pads, while the glass substrate consists of the pickup electrodes and the packaging pads. The microfabrication flow chart is illustrated in Figure 6 with the silicon-substrate process in the left column and the glass-substrate and packaging process in the right column. 

**Figure 7:**
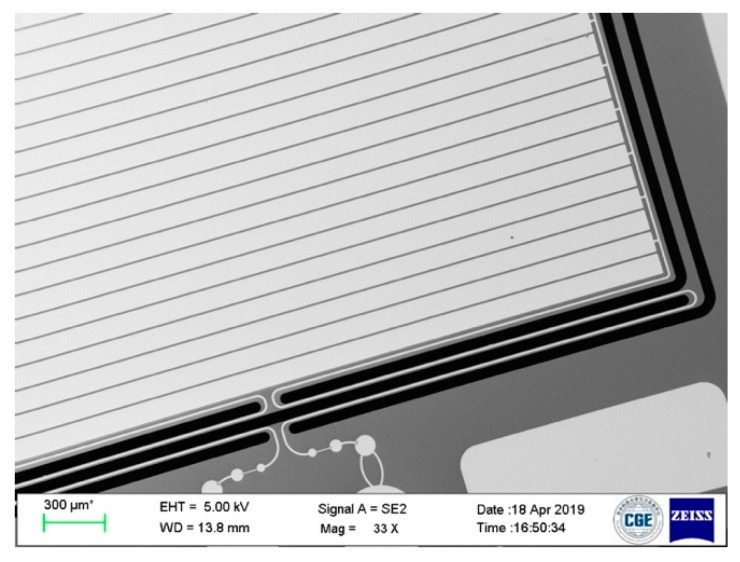
SEM image of the fabricated accelerometer chip.

Firstly, a 100 mm in diameter silicon wafer with double side oxidation was prepared. The backside oxidation layer was stripped by reactive ion etching (RIE), followed by the front side window patterning through photolithography. Then, the exposure pattern was etched by RIE to form an ohmic contact window. The ohmic contact was formed by AuSb (1% Sb, 150 nm)/NiCr (50 nm)/Au (200 nm) laminated metal annealing at 420 °C for 1 h. The resistivity of the ohmic contact was measured to be 10^−3^ Ω·cm^2^. In order to form the electrical ground for capacitive shielding and build the interconnections between different pads, the Metal 1 layer, which had a chrome adhesive layer of 20 nm in thickness and a gold layer of 200 nm in thickness, was deposited on the substrate surface by E-beam evaporation. Then, a layer of photosensitive polyimide (PSPI) was spin-coated and patterned. The PSPI layer was heat treated in an oven for imidization at 80 °C for 120 min, 150 °C for 60 min, 180 °C for 60 min, 250 °C for 60 min, and 350 °C for 60 min. In this case, the solidified PSPI features performed as the insulation layer between the Metal 1 features and the Metal 2 features. Then, the Metal 2 layer of Cr (20 nm)/Ni (80 nm)/Au (300 nm) was deposited by evaporation and patterned by lift-off technology to form the electrodes, signal traces, and packaging pads. In the following, a 10 µm thick AZ9260 photoresist was spin-coated and patterned to form the spring-mass structure mask. In the following, an aluminum layer of 200 nm in thickness was deposited on the backside of the wafer to alleviate the notching phenomena during the following through-silicon-wafer deep reactive ion etching (DRIE). A backing wafer was attached on the bottom of the device wafer by a 2 µm thick photoresist layer. Then, the Oxford Instrument inductively coupled plasma (ICP) system was used to conduct DRIE through-wafer-etching process. After photoresist and aluminum stripping, the silicon-based spring-mass structure with a thickness of 500 µm was released and free to move. Dicing-free technology was used to singulate each individual die from the wafer. The scanning electron microscope (SEM) image of the as-fabricated silicon layer is illustrated in Figure 7.

The upper glass cover plate of the proposed accelerometer was fabricated based on a 500 µm thick glass wafer. Firstly, the metal features of Cr (20 nm)/Ni (80 nm)/Au (300 nm) were fabricated by evaporation and lift-off technologies. Then, an aluminum layer of 200 nm in thickness was deposited and patterned to perform a hard mask for the following tin electroplating. A tin electroplating process at room temperature for 10 min was conducted to form the spacing and packaging features with a thickness of 15 µm. Then, the photoresist and aluminum layers were stripped. The glass wafer was then diced into individual dies.

**Figure 8:**
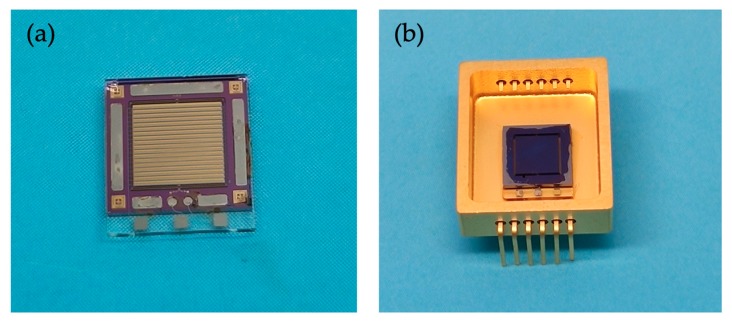
Diagrams of the accelerometer bare die and wire-bonding with the chip carrier: (**a**) the bare die of the MEMS accelerometer; (**b**) the customized chip carrier.

The proposed accelerometer chip is in sandwich structure. The silicon-based spring-mass layer and the glass-based upper cover plate were flip-chip packaged by AuSn thermocompression bonding at a temperature of 280 °C for 30 min. The lower glass cover plate, which was sand-blasted to form a cavity for the proof mass of the packaged accelerometer free to move, was glued by H70E on the backside of the silicon layer. The as-fabricated MEMS accelerometer chip (6.5 mm× 6.4 mm) is shown in Figure 8. The electrical input/output pads were wire-bonded by gold wires on a customized chip carrier.

## 4. Experiments

**Figure 9:**
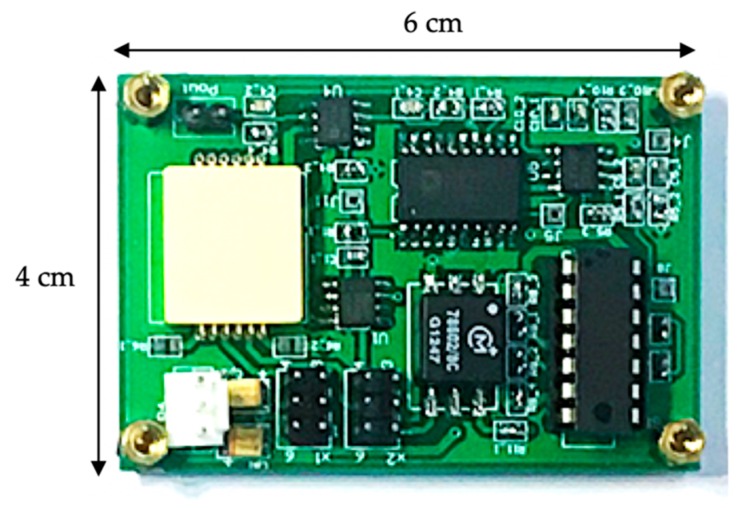
The accelerometer prototype on the signal conditioning circuit board.

In this section, the fabricated MEMS accelerometer prototype was connected on the signal conditioning circuit board, as illustrated in Figure 9, and several preliminary static experiments were conducted, including the scale factor calibration, the noise floor evaluation, and the bias instability evaluation.

**Figure 10:**
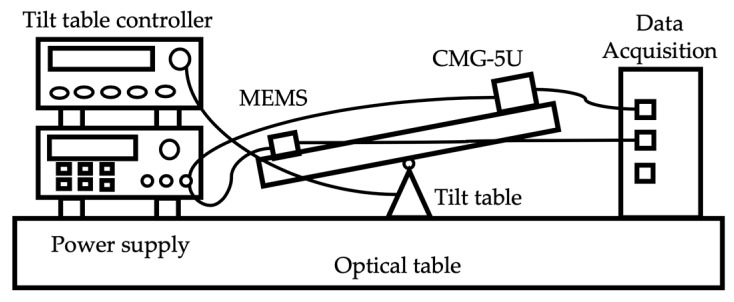
The calibration test diagram of the accelerometer.

In order to calibrate the scale factor of the proposed accelerometer prototype, a precise acceleration calibration system was set up, as shown in Figure 10. A commercial high-precision metal spring based single-axis accelerometer Guralp CMG-5U [29], which has a work range of ±2 g and a noise floor of about 0.2 µg/Hz^1/2^, was used as a reference sensor of the calibration system. Both the MEMS accelerometer prototype and the 5U accelerometer were fixed on the mounting surface of the tilt table which was driven by the controller with each step of 6.55°. Both the outputs of these two accelerometers were recorded by the National Instruments data acquisition system NI5922.

**Figure 11:**
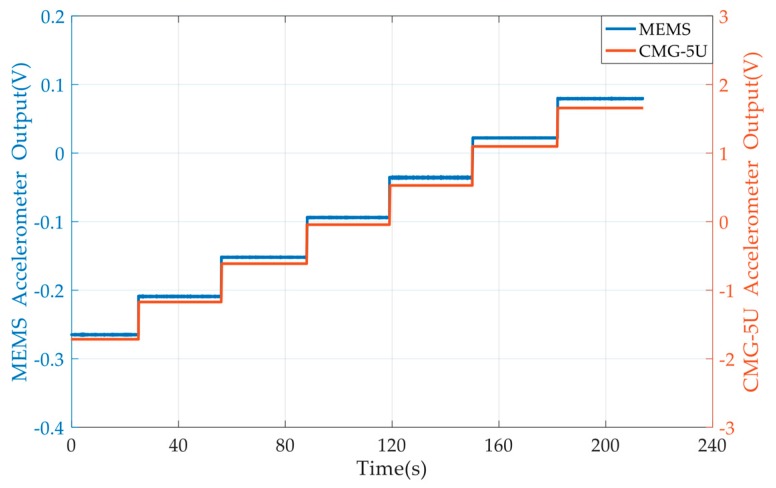
Outputs of both CMG-5U and the MEMS accelerometer under tilting.

When the tilt angle was zero, the sensitive axis of both accelerometers was about perpendicular to the gravitational acceleration vector; therefore, the outputs were both close to zero. When the tilt table was driven to different angles, the components of the gravitational acceleration were applied on both accelerometers. The outputs of both accelerometers were recorded, and one set of the measurement results is shown in Figure 11.

**Figure 12:**
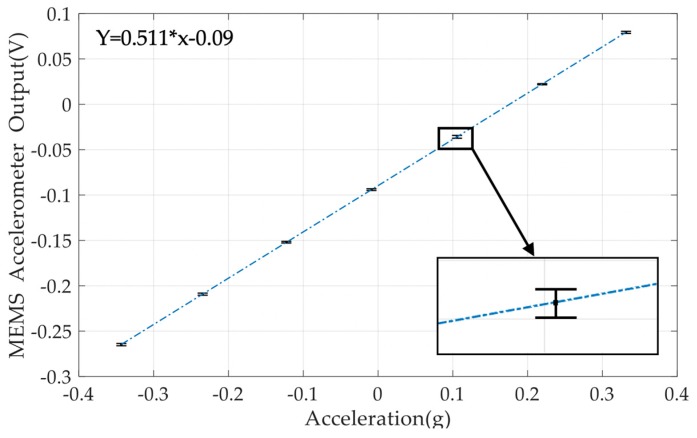
Scale factor calibration results of the MEMS accelerometer prototype.

Since the reference accelerometer was factory calibrated, the acceleration values at each tilt angle can be calculated by the known scale factor. Therefore, the voltage outputs of the MEMS accelerometer prototype in terms of accelerations can be plotted, as illustrated in Figure 12. By applying a linear fitting, the scale factor of the MEMS accelerometer was measured as 510 mV/g.

**Figure 13:**
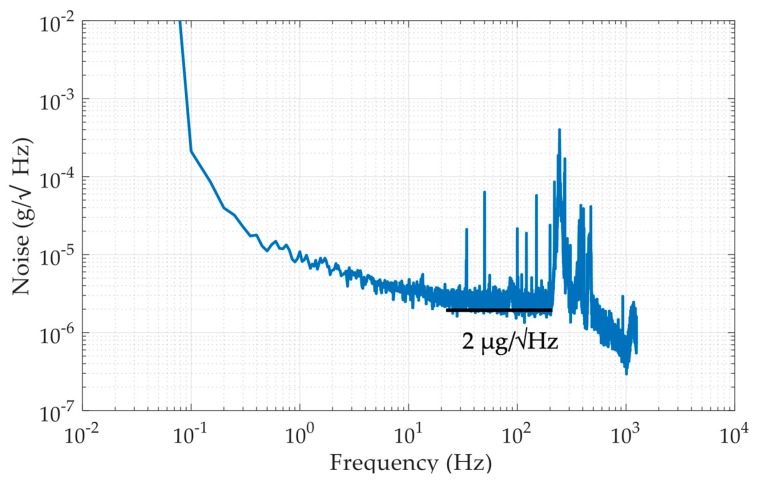
Results of the noise floor evaluation experiment.

The noise floor evaluation experiment and the bias instability evaluation experiment were carried out on the same site. The MEMS accelerometer prototype was mounted on an optical table and the output was recorded by the data acquisition system NI5922 with a sampling frequency of 2.5 kHz for 3 min. The power spectral density of the equivalent acceleration is plotted in Figure 13. It can be seen that the noise floor of the proposed MEMS accelerometer was 2 µg/Hz^1/2^ at 100 Hz. Apart from the fundamental frequency of about 270 Hz, other peaks were highly likely the resonant frequency of the optical table, the power frequency, and their harmonics. The low noise floor attributes to both the through-silicon-wafer-etching process for lowering down the thermal noise and enlarging the acceleration-to-displacement sensitivity and the periodic array area-variation capacitive displacement transducer for increasing the displacement-to-capacitance sensitivity.

**Figure 14:**
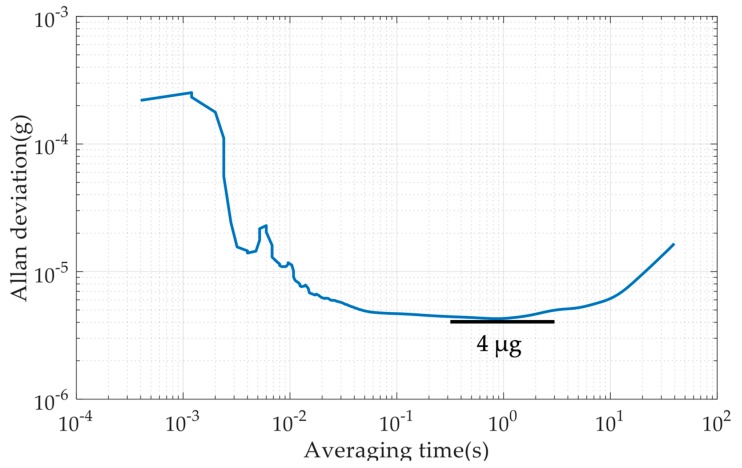
Results of the bias instability evaluation experiment.

The bias instability of the MEMS accelerometer was evaluated by the Allan deviation method with a sampling rate of 2.5 kHz. From Figure 14, it can be seen that the bias instability was 4 µg at the averaging time of 1 s.

Since the maximum voltage output was close to ±12 V and the scale factor of the MEMS accelerometer was 510 mV/g, the work range of the proposed accelerometer can be more than ±20 g. Several commercial off-the-shelf (COTS) MEMS capacitive accelerometers were compared with the proposed MEMS accelerometer in terms of the work range, the noise floor, the operation, and capacitive sensing technologies, as listed in Table 4. It can be seen that the area-variation based open-loop accelerometer proposed in this paper is reasonably better than the state-of-the-art COTS MEMS capacitive accelerometers. Therefore, this proposed MEMS accelerometer is attractive for inertial navigation, structural health monitoring, and tilt measurement applications.

## 5. Discussion

For the through-silicon-wafer DRIE process, a PlasmaPro NGP100 Estrelas by Oxford Instruments was used to run the Bosch process. The etching uniformity for a 100 mm wafer is within 3% as the manufactory claimed. However, it has been found that the undercut effect results in larger etching trenches and non-straight sidewall profiles during through-wafer-etching, which are the main contributions for the fabrication deviation. Several technologies were applied to solve these issues. One was the mask design compensation technology that used halo-masking technology to have all the etching trenches with the same width for good uniformity, and feature compensation, designing the folded beam width to 26 µm in the mask to obtain 16 µm beam width after etching. The second approach was recipe optimization and tuning: dummy runs were conducted to fine-tune the recipes for conformal etching profiles before each critical run. Based on these technologies, the etched profiles of the folded beam width were between 14 µm and 16 µm for the same batch and the cross-sectional profiles of the etching trenches were in bow shape. There were 25 dies produced and 6 dies were electrically tested, and the fundamental frequency deviations were about 20% ranging from 220 Hz to 280 Hz. In order to have comparable performance of fabricated dies from the same batch, fine trimming for sensing element geometry is generally required. There are at least two trimming technologies suitable for the proposed sensor. One is the laser trimming technology, and the other one is controlled post-etching technologies for silicon structure trimming. The post-silicon etching technology by reactive ion etching (RIE) will be applied to trim the sensors in the future. 

In order to obtain a low cross-sensitivity, both mechanical and electrical approaches were applied. Firstly, the sensor suspension was designed to have all spurious resonant modes far away from the fundamental mode. In this case, if an acceleration signal in an arbitrary direction is applied on the sensor, the displacements of the proof mass along other axes will be much smaller than that along the sensing axis. In addition, the gap of the upper and lower electrodes was set as 10 µm through packaging. Therefore, if a dynamic acceleration with a magnitude of 20 g is applied on the sensing direction *X*-axis, the displacement magnitude along *Z*-axis should be about 88 nm which is much smaller than the gap of 10 µm so that the proposed sensor has a low cross-sensitivity. Secondly, the periodic array area-variation capacitive displacement transducer design is intrinsically insensitive to differential gap variations. For the rotational modes about *X*-axis and *Y*-axis, the proof mass tilts so that the gaps of the matching electrodes are smaller at one side and larger at the other side. However, due to the periodic array configuration and long electrodes, the capacitive transducer is less sensitive to proof mass tilt. In the future, dynamic calibration will be applied to evaluate the cross-sensitivities of the sensors. 

## 6. Conclusions

In this paper, a micromachined open-loop area-variation capacitive accelerometer was designed, fabricated, and characterized. The through-silicon-wafer-etching process, connection beams, and the periodic array area-variation capacitive displacement transducer were applied to achieve high performance. Several experiments, including the tilt table calibration, the noise floor evaluation, and Allan deviation assessment, were conducted and the results showed that the proposed MEMS accelerometer had a scale factor of 510 mV/g, a noise floor of 2 µg/Hz^1/2^ at 100 Hz, and a bias instability of 4 µg at an averaging time of 1 s. Compared with the commercial MEMS accelerometers, the developed MEMS accelerometer in this paper has its superiority in noise floor and measurement range and is promising to be applied to industrial applications. The fabrication deviation and cross-sensitivities were discussed, followed by the possible reduction and evaluation approaches which will be performed in the future. 

## Figures and Tables

**Table 1:** Design parameters of the spring-mass structure.

Symbol	Parameters	Unit	Value
*L*_1_	Sensor length	mm	6.5
*W*_1_	Sensor width	mm	6.4
*L*_2_	Proof-mass length	mm	4.2
*W*_2_	Proof-mass width	mm	4
*L*_3_	Frame length	mm	1
*W*_3_	Frame width	mm	1
*l*	Folded beam length	mm	2
*l*_1_	Linkage length	µm	200
*l*_2_	Connection beam gap to frame	µm	50
*l*_3_	Connection beam width	µm	50
*l*_4_	Connection beam gap to mass	µm	50
*w*	Folded beam width	µm	16
*w*_1_	Folded beam gap	µm	50
*w*_2_	Linkage width	µm	50
*t*	Wafer thickness	µm	500
*f*_0_	Fundamental frequency	Hz	295.2

**Table 2:** Simulation results of the suspension systems with and without the connection beams.

Parameters	Sym.	With Connection Beams	Without Connection Beams
Fundamental frequency	*f_x_*	295.7 Hz	308.4 Hz
Rotational frequency about X-axis	*f_α_*	7368.9 Hz	5918.0 Hz
Out-of-plane translational frequency	*f_z_*	7489.3 Hz	4474.8 Hz
Rotational frequency about Y-axis	*f_β_*	13,523 Hz	7958.7 Hz
Rejection ratio	*f_α_/f_x_*	24.9	19.2
Rejection ratio	*f_z_/f_x_*	25.3	14.5
Rejection ratio	*f_β_/f_x_*	45.7	25.8
Cross-sensitivity in displacement	*z/x*	0.16%	0.48%

**Table 3:** Design parameters of the periodic array area-variation capacitive displacement transducer.

Symbol	Parameters	Unit	Value
a	Drive electrode width	µm	100
b	Pickup electrode width	µm	110
g_1_	Drive electrode separation	µm	10
g_2_	Pickup electrode separation	µm	110
*l_e_*	Electrode length	mm	4
d	Gap between the drive electrodes and pickup electrodes	µm	10
N	Number of periods	/	18

**Table 4:** Comparison of the proposed accelerometer with commercial off-the-shelf (COTS) MEMS capacitive accelerometers.

MEMS Cap. Acc.	Range (g)	Noise Floor (μg/Hz^1/2^)	Die size (mm^3^)	Operation	Cap. Sensing
MS9010 [30]	±10	18 @100 Hz	<8.9 × 8.9 × 3.2	Open-loop	Gap-variation
MAXL-OL-2020 [31]	±20	4 @100 Hz	<8.9 × 8.9 × 3.2	Open-loop	Gap-variation
ADXL 356B [32]	±20	80 @100 Hz	<6.0 × 6.0 × 2.2	Closed-loop	Gap-variation
This work	±20	2 @100 Hz	6.5 × 6.4 × 1.6	Open-loop	Area-variation
